# Simulation-based multiple-choice test assessment of clinical competence for large groups of medical students: a comparison of auscultation sound identification either with or without clinical context

**Published:** 2015-04-20

**Authors:** Diem Quyen Nguyen, Jean Victor Patenaude, Robert Gagnon, Benoit Deligne, Isabelle Bouthillier

**Affiliations:** 1 Centre d’Apprentissage des Aptitudes et des Habiletés, Cliniques-University of Montreal (in collaboration with CAE inc.)

## Abstract

**Background:**

Although simulation-based teaching is popular, high-fidelity, high-cost approaches may be unsuitable or unavailable for use with large groups. We designed a multiple-choice test for large groups of medical students to explore a low-cost approach in assessing clinical competence. We tested two different scenarios in assessing student’s ability to identify heart and lung sounds: by hearing the sounds alone, or in an enhanced scenario where sounds are incorporated into clinical vignettes to give clinical context.

**Method:**

The two-section test consists of multiple-choice questions with one best answer. In the first section, the student must identify 25 auscultation sounds from amongst a choice of 14 heart sounds and 11 lung-sounds. The second section integrates these same sounds into clinical vignettes to provide clinical context. Students must either identify the illness or the next clinical step, choosing from four possible answers. Performances of 859 students were evaluated.

**Results:**

The alpha coefficient of reliability is 0.54 and 0.76 respectively for the first and the second section. In the latter section there is significant difference between scores of first, second, fourth year students and residents, in contrast to the first-section scores.

**Conclusions:**

A multiple-choice test to assess clinical competence based on simulated auscultation sounds incorporated into clinical vignettes allows us to differentiate between training levels and seems to be a valid assessment method suitable for large-group format.

## Introduction

Simulation-based teaching is becoming very popular in the medical education community, and is strongly appreciated by medical students.^1^,^2^ Although studies of the impact of simulation on the acquisition of knowledge and development of clinical skills show encouraging results, data regarding its utility in curriculum development, including teaching strategy and impact on learning and evaluation methods, are yet to be obtained.^3^ As pointed out by Issenberg et al., simulation-based medical education (SBME) requires several important characteristics to be successful, including integration into the overall curriculum, respect of clinical variation, use of a valid simulator and clear outcome measurements.^4^ Learner assessment is a major area for research and development: “…*SBME outcome measurement is one of the greatest challenges now facing the field.”*^3^

Simulation-based assessment (SBA) frequently integrates simulators into a high-fidelity testing context, typically with objective structured clinical examination (OSCE)-type testing methods or highly sophisticated computerized case-based programs.^5^,^6^,^7^ This type of testing has strong face validity, but high cost limits its use to either a small number of assessment situations or to high-stake testing such as certification in internal medicine in Canada, or anesthesiology in Israel.^5^,^8^,^9^ Recently it was suggested that low-fidelity training (such as recognition of recorded heart sounds) could be comparable to expensive high-fidelity training (including the Harvey cardiopulmonary simulator-manikin).^10^ However, few studies have been conducted in this area. In 2006, Vukanovic-Criley et al. used computerized simulation-based testing to verify cardiac examination skills of a group of 860 students and staff, and reported a decline in examination skills amongst different groups of examinees as training level increases.^11^ Nevertheless, it is unclear if this is a true problem of clinical competence, or if this decline is due to an intrinsic validity problem of the exam format. Since then, there has been no other reported SBA study for very large groups of students to replicate these results.

Since 2008, medical student cohorts at the University of Montreal have increased to more than 250 students, and since 2009, SBME has been integrated into their curriculum. We created a multiple-choice question (MCQ) SBA since assessment should be part of this curriculum and financial limits do not allow for sophisticated high fidelity simulation-based testing. Traditional types of MCQ tests involving heart and lung sounds give a description of the auscultation sound in a clinical context, and ask students to choose an answer regarding diagnostic or therapeutic decisions. However, in real life the usual clinical approach consists of a patient giving a clinical context (e.g. acute chest pain), examination by a physician including heart auscultation (e.g. normal sounds could be found), a clinical diagnosis (e.g. angina), requests for diagnostic tests (e.g. an electrocardiogram) and a treatment decision (e.g. prescribing an aspirin). How can auscultation sounds be tested to verify clinical competence without giving away the auscultation diagnostic by describing it? Models for testing heart and lung auscultation sounds within clinical context for large samples of students are still lacking. It is unknown whether students who succeed in recognizing heart and lung sounds would be able to use them in clinical context to make clinical decisions.

In the present study we report results of our test that was created primarily to verify which of two formats a low-fidelity simulation (MP3 cardiac and pulmonary sounds from the University of Miami’s Harvey® The Cardiopulmonary Patient Simulator and Lecat’s ventriloscope) we should use in a multiple choice exam that can be given to large groups of students to assess their clinical competence. Should the test format involve only isolated recognition of auscultation sounds, or auscultation sounds incorporated into clinical vignettes (since it has been previously claimed that simulation incorporated into authentic clinical context has a greater impact on assessing clinical competence)?^12^ We hypothesized that although students’ ability to recognize auscultation sounds from simulators may decline as they continue in their clinical training and leave their formal studies behind, their ability to act upon them in a clinical context would not decline. As secondary objectives we examine test performance of students at different training levels according to their previous exposure to structured, simulation-based training, and also the acceptability of both test formats.

## Methods

Since the primary aim of the test is to assess clinical competence using simulation-based multiple-choice questions, its validity will be mainly supported through its ability to differentiate students’ training levels and their previous exposure to simulation. To be able to compare both test formats, performance in auscultation-sound recognition will be compared to performance in solving clinical questions when these same sounds are incorporated into a clinical context.

### Participants

To study whether the test can assess clinical competence, the performances of five groups of medical students at different levels of training were evaluated. The first-year group of students have no structured training in simulation or any clinical training. Second-year students have structured simulation-based training in auscultation of the heart and the lungs, but have little clinical experience. Their training consists of six weekly workshops of one-hour duration for groups of eight students. The workshop begins with a short theoretical demonstration, followed by students listening to different heart sounds and murmurs using recordings from either the Harvey simulator or Lecat’s ventriloscope under the supervision of highly-trained clinical physicians. The content of the workshop includes normal sounds and pathological findings, systolic and diastolic murmurs, as well as lung sounds including crackles, high-pitched rhonchi and rubs. Students who want further practice are able to sign up for independent learning in the simulation center. During second year, medical students also have problem-based study sessions in cardiology and respirology, and review heart and lung auscultation sounds at the bedside with both cardiologists and lung specialists. Third-year students are six months into their clerkship and have a beginner’s level of clinical experience. The fourth-year students have both informal simulation training and 18 months of clinical experience. During the third and fourth years, students have an eight-week internal medicine rotation, and heart and lung auscultation teaching are mostly done as bedside demonstration. Finally, the first-year residents (PGY1) in internal medicine have a more advanced level of clinical experience and have had informal simulation training two months before the test (a one-hour heart sound demonstration with the Harvey simulator given by a cardiologist and another one-hour workshop reviewing lung sound auscultation with pulmonary specialists using Lecat’s ventriloscope). They have also had cardiology and pulmonary clinical rotations, with heart and lung auscultation mainly taught at patients’ bedside.

All medical students from the first to fourth year, as well as first-year internal medicine residents, were invited to take a simulation-based test in January 2011. Learners at more advanced levels of training were not invited due to their small number. The scores of this exam did not count in the students’ evaluation. The invitation was sent to each student via internet from the faculty education office; the researchers did not have access to individual email addresses.

Each participant signed their informed consent on their exam day, and only the results of participating students were analyzed. To ensure the confidentiality of the results, each student was assigned a numeric code and the results were sent with these codes to the principal investigator. Only the education office had the students’ name and code list. Approval of the research protocol was obtained for the study through the Educational Review Board and the Research Ethics Board of the University of Montreal.

### Simulation-based exam

Our test was a two-part multiple-choice exam with one best answer. The first section consists of 25 questions with a 14-choice heart-sound menu and an 11-choice lung-sound menu. Participants identified basic heart and lung sounds (Table 1). The exam content includes all the heart and lung sounds of the simulation-based training curricular objectives of the medical student’s level. These sounds were converted into MP3 format from the original sounds of Lecat’s ventriloscope and from the Harvey© Cardiopulmonary Patient Simulator (provided by the Michael Gordon Center of Research in Medical Education) to replicate the sounds that are used during students’ formal training.

The second section of the exam consists of integrating these same heart and lung sounds into 25 clinical vignettes. Questions with four possible answers either ask students to recognize the illness possibly related to these sounds, or to propose the next clinical step, providing clinical context and findings based on these heart or lung sounds. Clinical contexts included in the vignettes are either concordant or discordant to clinical situations and are randomly distributed to auscultation sounds to decrease cueing effect.^18^ Examples of both clinical situations are illustrated in Table 1.

The test content (clinical vignettes and correct answers) was reviewed independently by two internal medicine residents, two general internists with at least 15 years of clinical experience, and the program director (to ensure the comprehensiveness of the questions, its clinical pertinence and its appropriate level of difficulty).

The exam was given in a 1345-seat auditorium. Sounds were transmitted by two Gentner TX-37A speakers from US-based Starin Company. At the entrance, each student was provided with answer-sheets, pencils and the Procom audio Rex-7 receptor (the same audio system usually used in large group conferences). Diagrams of the Harvey simulator showing the origin of the sounds along with the pertaining question were projected with PowerPoint. Each question was repeated twice, lasted 30 seconds each time, and there was no possibility to go back to a previous question. At the beginning of the test, examples of normal sounds were given. Students were told that the same sounds could be used more than once, and that these sounds were randomly distributed throughout the test. There were two sessions in the same day, each with 500 students separated by an empty seat from one another. The same test was given to all participating students.

At the end of the test, a questionnaire was given to all participating students to obtain their demographic data and previous experience with simulators, as well as their opinion of the exam and the use of simulation as part of their training. A five-point Likert scale, 1 being defined as “strongly disagree” and 5 being “strongly agree,” was used to get students opinions. To verify their ability to self-assess, a question about their performance in diastolic and systolic heart murmur was added to the questionnaire, and then compared to their test score with these same types of heart murmurs.

### Data analysis

Descriptive statistics (mean, standard deviation and frequencies) were obtained for each section of the test for the five training levels. The total score represented the sum of scores for all 50 questions. Score 1–25 was the sum of the first section (simple recognition of sounds), and score 26–50 was the sum of the second section (representing performance of recognition and interpretation of the auscultation sounds in a clinical context). A paired t-test was used to compare the mean of the scores between the test sections. Test items were analyzed with one-way analysis of variance (ANOVA), and a *post-hoc* Scheffé test was used to verify the difference between training levels. An ANOVA test of linearity was also done to verify the improvement between each level of training. Reliability of the test was obtained using the Cronbach alpha coefficient.

To compare the performance in recognizing auscultation sounds and then recognizing these same sounds in a clinical context, the Pearson correlation coefficient (*r*) for each pair of auscultation sounds was obtained for each level of training. Only a statistically significant positive correlation was considered meaningful.

The satisfaction questionnaire was analyzed with descriptive statistics. SPSS version 17.0 (SPSS, Inc., Chicago, IL, USA) was used for all statistical analysis and *p*<0.05 was considered to be significant.

## Results

From a population of 1037 students (255 from 1^st^ year, 280 from 2^nd^ year, 216 from 3^rd^ year, 246 from 4^th^ year and 40 PGY1), 859 consented to give access to their test scores for analysis. Their demographic data, reported experience with simulation-based training and their clinical training in cardiopulmonary auscultation are reported in Table 2. Most of the students reported having had between one and six hours of formal teaching in heart and lung auscultation during their clinical training, 50% having had three to five hours. However, 70.2% of junior residents reported having had between six and 15 teaching hours. Students at different levels admitted that they seldom practiced their heart and lung auscultation with U-Medic that is made available to them on a voluntary, self-study basis.

### Test performance

The Cronbach alpha coefficient of reliability for the whole exam is 0.77. Reliability analysis for each exam section shows that the first section, where only simple recognition of cardiopulmonary sounds is required, does not achieve a high reliability coefficient (*a* = 0.54) in contrast to the second part (*a* = 0.76).

**Figure 1:**
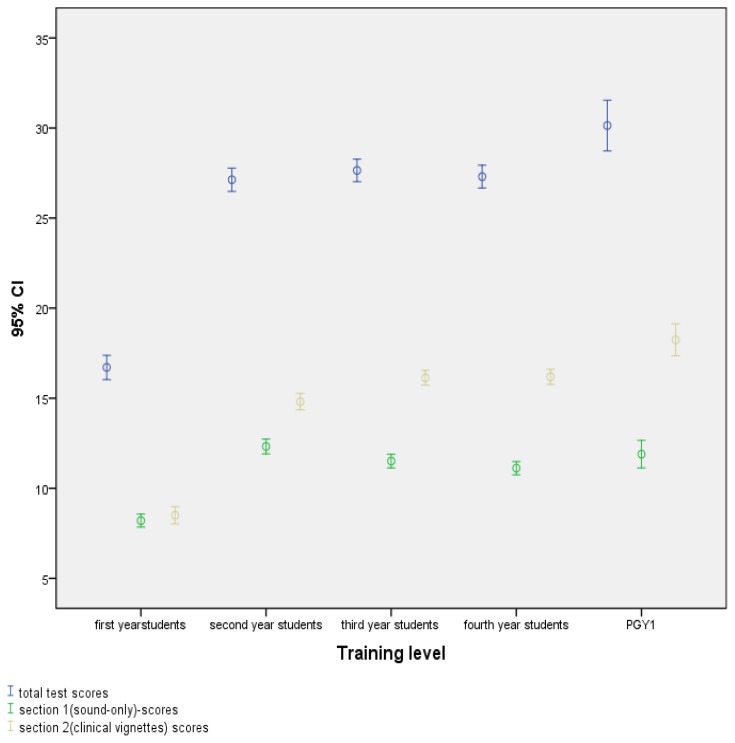
Test scores according to training level

An ANOVA test for linearity confirmed a significant linear and positive progression between different levels of training on the total exam (F(4,854) = 204.82, p < .001) (Figure 1). Test performance for the whole exam and per section is shown in Table 3. Total scores increase between four distinct levels of clinical experience: from novice first-year students to PGY1 with much more clinical training. However, detailed analysis shows that performance according to student level of training in both sections differs greatly. For the sound-only recognition section, while the ANOVA was significant, the only statistically significant difference was between the first-year students and all the other levels (F(4,854) = 64.99, p<.001). *Post-hoc* Scheffé test (See Table 3) indicated that the mean score for the first year students in this section (M=8.2, SD=2.5) is significantly different between other years’ student scores (M=12.3,SD=2.9 for the second year students, M=11.5, SD=2.7 for the third year students, and M=11.1,SD=2.7 for the fourth year students). However, there were no statistically significant differences between these last three levels of training. In contrast, when those same sounds are incorporated into clinical vignettes there is a steady improvement as training levels increase (F(4,854)=214.16, p<.001); post hoc Scheffé test results show significant difference between 1^st^ year and second year score (M=8.5,SD=3.3 vs. M=14.8,SD=3.3,p<0.001), second and third year score (M=14.8,SD=3.3 vs M=16.1,SD=2.9, p<0.001), as well as fourth year and PGY1 score (M=16.1, SD=3.1 vs. M=18.2,SD=2.7,p <0.001). Although the positive differences in scores with increasing training are small, they are statistically significant between student groups differing in their level of clinical experience (between first, second, and the last two years). This difference remains statistically significant between students in the last year of the medical school program and the first year of residency, where, in Montreal, the training is almost exclusively clinical.

Performance with different pairs of sounds (for example, a question about recognizing a heart sound, and another question concerning a clinical vignette with a patient having that same sound) according to students’ training level is illustrated in Table 4.

The number of auscultation pairs having a positive correlation is small: among 25 pairs of auscultation sounds, only 8 pairs with the second and third-year students have a positive and significant correlation. These students are the ones who had repeated formal training with simulators. The PGY1 has a smaller number of positively correlated pairs. As previously described, these students only have two hours of heart and lung sound demonstration. No consistent pattern could be found, and it seems that performance in recognizing isolated auscultation sounds does not correlate with performance in acting upon these same sounds in a clinical context.

### Satisfaction study

To verify students’ ability to self-assess, and thus verify the reliability of their answers with the satisfaction questionnaire, a question about their perception of performance regarding diastolic and systolic murmurs was added. According to 68.4% students, diastolic murmurs are more difficult to recognize. Their performance shows that indeed, the mean score with diastolic is significantly lower than with systolic murmurs.

As expected, all the students found the exam to be difficult. However, they seemed to appreciate this type of test: the advanced-level students found that the exam was adjusted to their level of training. A very high proportion of the students (87.8%) either slightly or totally disagreed that the first section is easier than the second section, and most students (95.2%) reported that adding clinical vignettes helped them to focus on the auscultation sounds. Only 26.2% of first-year students totally agreed with this latter point, as opposed to 89.2% of PGY1.

## Discussion

Our findings seem to corroborate Shuwirth’s opinion that any assessment should respect authentic context to achieve validity: results in the first section where simple recognition of auscultation sounds is required do not show a significant difference among training levels. In the second section, where simulation-based auscultation sounds are integrated into clinical context, there is a small but statistically significant difference between most cohorts of students, even first and second year, second and third year, as well as between fourth year and first-year residency. One possible explanation for no significant difference between the third and the fourth-year students could be that at our university, the third and the fourth year are mainly clinical rotations without a compulsory curriculum, providing that at the end of the two years all basic rotations (internal medicine, family medicine, surgery, obstetric and gynaecology, paediatrics, psychiatry, anaesthesia, ophthalmology as well as rural medicine) are completed. Students’ study paths could be sufficiently heterogeneous that no difference in clinical competence could be detected.

Our study seems to confirm the tendency towards performance improvement found in previous studies when clinical context is added: in one study where residents have to identify a heart sound and murmur, adding clinical context improves the number of residents who succeed from 74% to 90%.^13^,^19^ It also seems that depending on either concordant or discordant clinical context, performance could be biased and diagnostic accuracy affected. Although adding clinical context may cue students to make correct responses, the impact and how to address this potential bias is unclear. Both concordant and discordant clinical situations are deliberately used and randomly distributed in our test to decrease any potential cueing effect.^18^ Although the MCQ exam could only superficially test clinical reasoning, our data adds further evidence to support the theory that a clinical situation should be added to simulation-based assessment whenever clinical competence is evaluated.^12^ Subsequent studies should investigate the frequency of each type of clinical context to be included in an SBA-MCQ test to best differentiate clinical experience levels.

Our data also seem to show that, within the limits of MCQ testing, total mean scores in cardiac and lung auscultation skills of our medical students vary between 33.4% for the first year to 60.2% for the PGY1 level. This seems to highlight an area of weakness frequently encountered, as has been repeatedly reported previously.^7^,^14^,^15^,^18^,^20^ However, it is reassuring to observe an improvement of their competence in interpreting heart and lung sounds in a clinical context. Additional studies would be interesting to help explain why some students recognized isolated heart and lung sounds, but failed to act upon these same auscultation sounds when put into clinical context.

Although the number of auscultation pairs having a positive correlation is small, and the correlation coefficient is weak, one could wonder if formal training with high-fidelity simulators has some impact on the ability to recognize auscultation sounds when using them in a clinical context as pointed out by other studies showing the benefit of SBME with deliberate practice.^16^,^17^ Could this explain the very low scores of our first-year students who have not yet had any formal training? The number of positive and significant correlations is highest with both second- and third-year students, where formal and repeated training with simulators had been incorporated into their curriculum. These findings are yet to be corroborated by other studies, since this is the first time that such results are noticed in this type of testing. Meanwhile, these results could help program directors to plan their teaching of cardiac and pulmonary physical examinations and to take into account the need of repeated focused training especially for students with learning difficulties.

Students and residents generally consider this exam format to be difficult, but as their level of training increases they seem to appreciate the appropriateness of this type of assessment, and seem to prefer clinical vignettes over isolated recognition of heart and lung sounds. To verify their honesty in answering the appreciation form (since it was filled out at the end of the exam and could be influenced by their performance) we validate their ability of self-assessment by asking them about their performance with diastolic and systolic murmurs. Most of the students find that diastolic murmurs are much more difficult to recognize. Indeed, their performance with diastolic murmurs is significantly worse than with systolic murmurs. It seems that students have a realistic view about SBA-MCQ-type exams: they are difficult, but they seem to be adjusted to their training level

This is the first study addressing the issue of low-fidelity simulation-based assessment by multiple-choice exam for a very large group of medical students with comparison of performance between different training levels. Our encouraging results should be used with caution however, as our study was conducted within a single institution, and with only a small group of PGY 1. These results may also have been influenced by the fact that our teaching program includes theoretical lectures in the second year, as well as integration of clinical experience when physical examination skills are taught in the second year. To minimize the potential impact of the moment when the students were tested and the timing in their curriculum, all the students had their test on the same day at mid-term (at the beginning of January). At this time, all the second year-students have had their formal teaching in cardiology and respirology, and half of the third year students have had their internal medicine rotation. The fourth year students have all completed their internal medicine rotation in their third year. The lack of difference between the third and the fourth year could result from such heterogeneous clinical exposure. It would be interesting to follow these student cohorts to compare their later performance in actual clinical settings. Prospective and multi-university studies with SBA would help to determine the comparability of our findings. It would also be interesting to test this concept in other non-physician groups such as nurses or physician assistants.

## Conclusion

Our study shows that a multiple-choice question test using audio sounds from a high-fidelity manikin combined with clinical vignette assessment, compared with recognition of auscultation sounds on their own, seems to provide reliable and valid data, and enables us to better differentiate among levels of clinical competence. Furthermore, good acceptability of the test format by students at advanced training levels suggests that MCQ-SBE is a reasonable test method in a low-stake assessment for large groups of students.

## Figures and Tables

**Table 1:** Illustration of section 1 and section 2 SBA multiple choice questions

Section 1: Heart sound choice menu	Section 1: Lung sound choice menu
Normal auscultation S3 S4 S3 & S4 Benign murmur Pericardial rub Pulmonary regurgitation Mitral regurgitation Mitral stenosis Aortic regurgitation Aortic stenosis Tricuspid regurgitation Tricuspid stenosis Aortic sclerosis	Fine crackles Coarse crackles Diffuse crackles Humid crackles Wheezing Decrease lung sounds Pleural rub Rhonchi Crackles and wheezing Stridor Bronchial alveolar breathing Normal vesicular auscultation
Section 2: Examples of clinical vignettes	
1- Concordant clinical situation: **Stem**: A patient presenting to the emergency room for prolonged cough with greenish sputum. **Harvey lung sound**: crackles on the left side, normal lung sound on the right **Question**: what would the chest X-Ray findings be? normal, lobar consolidation, bilateral basal infiltrates left-side effusion.	
**2-** Discordant clinical situation: **Stem**: A patient presents at your private office for an annual check-up. **Harvey lung sound**: Benign murmur at the mitral location **Question**: according to you, this murmur requires a cardiac echography results from a congenital malformation is benign requires annual medical surveillance	

**Table 2:** Demographic data of participating students

Training Level	n	Sex		Previous training with simulators* (%)	Previous teaching in auscultation† (%)	Self-training with U-MEDIC‡ (%)
		♂ (%)	♀ (%)			
**1st year**	195	33.3	65.6	0	1	1
**2nd year**	208	33.2	66.8	90.4	39.8	2.4
**3rd year**	204	29.9	70.1	86.8	22.6	3.6
**4th year**	215	36.3	63.7	78.1	20.5	7.0
**PGY1**	37	29.7	70.3	54.0	70.2	2.7

* Previous formal training with cardiopulmonary simulators (during the last 12 months as reported by students).

† Previous structured teaching in heart and lung auscultation during clinical rotation (having more than 6 hours) as reported by students.

‡ Self-training with U-MEDIC, a Multimedia Computer Curriculum where bedside skills are taught through video demonstrations of Harvey^®^, The Cardiopulmonary Patient Simulator, a full-size manikin that simulates the physical findings of essentially any cardiac disease (% students having used this software).

**Table 3:** Students’ scores in different test sections and according to their training level

						ANOVA	Post- hoc Scheffé test			
Test	1^st^ year	2^nd^ year	3^rd^ year	4^th^ year	PGY1	*p*	*p* 1 vs. 2	*p* 2 vs. 3	*p* 3 vs. 4	*p* 4 vs. PG1
**Total score mean (SD)** *	16.7 (4.7)	27.1 (4.7)	27.6 (4.5)	27.3 (4.7)	30.1 (4.2)	F(4,854)=204.82 *p<0.001*	<0.001	0.26	0.45	0.001
**Sounds alone mean (SD)**†	8.2 (2.5)	12.3 (2.9)	11.5 (2.7)	11.1 (2.7)	11.9 (2.2)	F(4,854)= 64.99 *p<0.001*	<0.001	0.062	0.14	0.104
**Clinical vignette mean (SD)**‡	8.5 (3.3)	14.8 (3.3)	16.1 (2.9)	16.1 (3.1)	18.2 (2.7)	F(4,854)=214.16 *p<0.001*	<0.001	<0.001	0.87	<0.001

* calculated over the total 50 questions;

† first section: sound-only recognition, calculated over the 25 sound- recognition questions;

‡ second section: sounds and clinical vignettes, calculated over the 25 questions with sounds integrated into clinical vignettes

**Table 4:** Correlation between the two heart- and lung-sound test sections according to training level

Type	Sound nature	Simulator	1^st^ year	2^nd^ year	3rd year	4th year	PGY1
**Heart sounds**	normal	Harvey	0.03	−0.05	0.11	−0.02	0.14
	S3	Harvey	0.08	0.21*	0.24*	0.11	0.10
	S4	Harvey	0.24*	0.13	0.31*	0.32*	0.19
	Pericardial rub	Harvey	0.09	0.29*	0.23*	0.38*	0.36*
	Gallop (both S3&S4)	Harvey	0.04	−0.07	0.01	0.04	−0.12

**Systolic murmurs**	Benign	Harvey	0.03	0.06	0.10*	0.01	0.10
	Aortic valve sclerosis	Harvey	0.08	−0.02	0.18	0.04	0.62*
	Aortic stenosis	Harvey	0.34*	0.03	0.18*	0.14*	0.28*
	Mitral regurgitation	Harvey	0.09	0.25*	0.11	−0.01	−0.05
	Tricuspid regurgitation	Harvey	0.05	0.03	0.09	0.02	0.09

**Diastolic murmurs**	Aortic regurgitation	Harvey	0.04	−0.02	0.04	0.07	0.06
	Pulmonary regurgitation	Harvey	−0.04	−0.04	0.11	0.14*	0.32*
	Mitral stenosis	Harvey	−0.06	0.16*	0.20*	0.12	0.01

**Lung**	Normal vesicular breath sound	Harvey	−0.03	0.01	−0.01	0.04	0.60*
	Absent/decreased lung sounds	Ventriloscope	−0.02	−0.02	−0.03	0.12	−0.17
	Fine rales	Ventriloscope	0.13	0.08	−0.01	0.06	−0.14
	Coarse crackles	Ventriloscope	0.13	0.08	0.07	0.04	0.11
	Diffuse crackles	Harvey	0.11	0.16*	0.13	0.07	0.19
	Humid crackles	Ventriloscope	0.14	−0.02	−0.05	−0.06	0.13
	Bronchoalveolar sound	Ventriloscope	0.14	−0.02	−0.049	−0.06	0.13
	Wheeze	Ventriloscope	0.04	0.12*	0.03	0.04	0.15
	Stridor	Ventriloscope	0.02	−0.25*	−0.21*	−0.25*	−0.35*
	Wheeze and crackles	Harvey	0.04	−0.02	0.17*	0.19*	0.18
	Pleural rub	Harvey	0.027	0.19*	0.24*	0.02	0.14
	Rhonchi	Ventriloscope	0.11	0.16*	0.08	0.03	0.17

* p < 0.05
